# Total Flavonoids from *Chimonanthus nitens* Oliv. Leaves Ameliorate HFD-Induced NAFLD by Regulating the Gut–Liver Axis in Mice

**DOI:** 10.3390/foods11142169

**Published:** 2022-07-21

**Authors:** Wenya Meng, Zitong Zhao, Lingli Chen, Suyun Lin, Yang Zhang, Jing He, Kehui Ouyang, Wenjun Wang

**Affiliations:** 1 Jiangxi Key Laboratory of Natural Products and Functional Food, College of Food Science and Engineering, Jiangxi Agricultural University, Nanchang 330045, China; mwy19970909@163.com (W.M.); zzt1994@jxau.edu.cn (Z.Z.); chenlingli89@163.com (L.C.); ncusklinsuyun@163.com (S.L.); jiushixiaoyang77@sina.com (Y.Z.); hj1903375065@sina.com (J.H.); 2 College of Animal Science and Technology, Jiangxi Agricultural University, Nanchang 330045, China

**Keywords:** *Chimonanthus nitens* Oliv. leaves, flavonoids, non-alcoholic fatty liver disease, inflammation, gut–liver axis

## Abstract

Non-alcoholic fatty liver disease (NAFLD) is one of the chronic liver diseases with high incidence in the world. This study aimed to investigate whether total flavonoids from *Chimonanthus nitens* Oliv. leaves (TFC) can ameliorate NAFLD. Herein, a high-fat diet (HFD)-induced NAFLD mice model was established, and TFC was administered orally. The results showed that TFC reduced the body weight and liver index and decreased the serum and hepatic levels of triglyceride (TG) and total cholesterol (TC). TFC significantly reduced the activity of liver functional transaminase. Aspartate aminotransferase (AST) and alanine aminotransferase (ALT) decreased by 34.61% and 39.57% in serum and 22.46% and 40.86% in the liver, respectively. TFC regulated the activities of oxidative-stress-related enzymes and upregulated the protein expression of nuclear factor E2-related factor (Nrf2)/heme oxygenase (HO-1) pathway in NAFLD mice, and the activities of total superoxide dismutase (T-SOD) and glutathione peroxidase (GSH-PX) in serum were increased by 89.76% and 141.77%, respectively. In addition, TFC reduced the levels of free fatty acids (FFA), endotoxin (ET), and related inflammatory factors in mouse liver tissue and downregulated the expression of proteins associated with inflammatory pathways. After TFC treatment, the levels of tumor necrosis factor-α (TNF-α), interleukin (IL)-6 and IL-1β in the liver tissues of NAFLD mice were downregulated by 67.10%, 66.56%, and 61.45%, respectively. Finally, TFC reduced liver fat deposition, oxidative stress, and inflammatory response to repair liver damage and alleviate NAFLD. Further studies showed that TFC regulated the expression of intestinal-barrier-related genes and improved the composition of gut microbiota. Therefore, TFC reduced liver inflammation and restored intestinal homeostasis by regulating the gut–liver axis. Overall, our findings revealed a novel function of TFC as a promising prophylactic for the treatment of NAFLD.

## 1. Introduction

Non-alcoholic fatty liver disease (NAFLD) is a common chronic liver disease with a high incidence of up to 25% all over the world [1]. NAFLD is one of the clinicopathological syndromes of fatty deposits in the liver in the absence of excessive alcohol consumption [2]. Simple steatosis, an early symptom of NAFLD, can later progress to non-alcoholic steatohepatitis (NASH), cirrhosis, and even hepatocellular carcinoma [3]. In addition, NAFLD is usually closely related to obesity, hypertension, hyperlipidemia, diabetes, and cardiovascular diseases [4]. 

There are many factors influencing the incidence of NAFLD, such as environmental factors, diet, and lifestyle. Furthermore, a high-fat diet (HFD) has been confirmed to cause obesity and hyperlipoidemia, which are closely related to the occurrence and development of NAFLD [5,6]. However, the pathogenesis of NAFLD is a complex serial process which is still incompletely understood. The “two-hit” hypothesis is the most widely accepted theory. The “first hit” refers to excess free fatty acids (FFAs) in the liver, leading to fat deposition. The fat deposition causes a “second hit” by stimulating the liver to produce too much of the reactive oxygen species, generating oxidative stress and liver cell damage [7]. Meanwhile, excessive FFAs can also stimulate inflammatory macrophages to release inflammatory factors, thus further activating hepatic stellate cells and aggravating liver damage [8]. In recent years, with the deepening of research, it has been found that there are other mechanisms affecting the occurrence and development of NAFLD, so the theory of “multi-hit” has been proposed [9]. However, both the “two-hit” hypothesis and the “multi-hit” theory prove that metabolic disorders, oxidative stress, and inflammatory response undoubtedly play an important role in the pathogenesis of NAFLD.

In addition, a growing number of studies have reported that intestinal health is related to NAFLD in recent years [10]. Bacteria and metabolites in the gut can cross the intestinal barrier and enter the liver through the portal vein. Intestinal barriers include chemical, mechanical, immune, and biological barriers [11]. When the intestinal barrier is destroyed and the gut microbiota is disturbed, more harmful bacteria and metabolites, such as endotoxins (ETs), may enter the liver through blood circulation, prompting the liver to release inflammatory factors, and then lead to further damage of intestinal organs [12,13,14]. NAFLD can be alleviated by regulating microbial balance and mucosal barrier [5,15]. Therefore, it is worth researching the NAFLD-alleviating effects by regulating the gut–liver axis. 

Currently, the main drugs used to treat NAFLD are insulin sensitizers; hypolipidemic drug; and antioxidants, such as polyene phosphocholine capsules (PPC), vitamin D, and metformin [16,17]. However, long-term use of these drugs can lead to weight gain, limb swelling, and other side effects [18,19]. Therefore, looking for drug substitutes with low side effects has become the focus of research worldwide, and some natural active ingredients in plants have been shown to alleviate NAFLD. For example, lupine peptide can repair HFD-induced liver injury by altering hepatic lipid signaling pathway [20], polyphenols of Raw Bowl tea can prevent NAFLD by regulating intestinal function in mice [12], and the water extract of broccoli can increase lipolysis and regulate M1/M2 polarization of macrophages to ameliorate NAFLD [21].

*Chimonanthus nitens* Oliv. (CNO), a unique Chinese plant belonging to *Chimonanthus*, is mainly distributed in Southern China [22]. Its leaves are consumed as a traditional Chinese tea, which is also called Golden tea or Shi-Liang tea. In previous studies, we have demonstrated that CNO can enhance antioxidant capacity, regulate glucolipid metabolism, possess antibacterial properties, and modulate the immune system [23,24,25]. However, there are few reports about the ameliorative effect of CNO leaves on NAFLD. 

The main purpose of this study was to evaluate the ameliorative effect of total flavonoids from CNO leaves (TFC) on HFD-induced NAFLD in mice and explore its therapeutic mechanism. Our study will provide a theoretical basis for the rational use of TFC to prevent and improve NAFLD in the future.

## 2. Materials and Methods

### 2.1. Materials

CNO leaves were purchased from Lishui, Zhejiang Province, China, and identified by Prof. Zhiyong Zhang (School of Agricultural Science, Jiangxi Agricultural University). Flavonoid standards (>98%) for HPLC were purchased from Solarbio (Beijing, China). The diagnostic kits used for investigation of TG, TC, AST, ALT, malondialdehyde (MDA), T-SOD, and GSH-PX were purchased from Nanjing JianCheng Bioengineering Institute (Nanjing, China). ELISA kits for TNF-α, IL-6, IL-1β, FFA, and ET were purchased from Boster Biological Engineering Co., LTD. (Wuhan, China). All other reagents (analytical grade) used in this study were purchased from local suppliers. 

### 2.2. Preparation of TFC

The preparation of TFC was based on Chen’s method and modified slightly [23]. The dried CNO leaves were pulverized and sieved for ultrasonic assisted extraction with 40% alcohol. The extraction conditions were as follows: ethanol concentration was 60%, ultrasonic time was 60 min, ultrasonic power was 1500 W, temperature was 40 °C, and solid–liquid ratio was 20 g/mL. The crude extract was adsorbed with polyamide resin, and gradient elution was carried out by using water, 30% ethanol, and 60% ethanol in sequence. Finally, 60% ethanol eluent was collected and freeze-dried into powder. The total flavonoids content in the powder was measured to be 75.33%, and we defined the powder as TFC.

### 2.3. LC–MS and HPLC Analysis

The analysis conditions of LC–MS refer to our previous research methods, with some modifications [23]. The corresponding mobile phase consisted of the 0.2% acetic acid (CH_3_COOH) in aqueous solution (A) and acetonitrile (B). The gradient of the mobile phase was as follows: 0–15 min, 5–40% A; 15–30 min, 40–95% A; 30–32 min, 95% A; and 32–35 min, 95–5% A. LC–MS conditions are as follows: anion mode; sheath gas pressure, 50 psi; spray voltage, 4000 V; vaporizer temperature, 500 °C. 

Flavonoids were quantified by the Agilent HPLC system, which was equipped with UV–Vis detector and C18 (250 mm × 4.6 mm × 5 μm, Waters, Ireland) column. HPLC conditions were the same as the LC–MS conditions described above, except that the flow rate was changed to 1 mL/min. The UV detection wavelength was set at 254 nm.

### 2.4. Antioxidant Capacity of TFC In Vitro

The 1,1-Diphenyl-2-picrylhydrazyl (DPPH) radical (DPPH•), 2,2′-Azino-bis (3-ethylbenzothiazoline-6-sulfonic acid (ABTS) radical (ABTS+), scavenging activities, and ferric reducing power of TFC were measured according to a reported method, with proper modification [26]. 

### 2.5. Animals and Experiment Design

Six-week-old male Kunming mice (20 ± 2 g) were purchased from Hunan SJA Laboratory Animal Co., Ltd. (SCXK (Xiang) 2016-0002, Changsha, China). All the mice were acclimatized for 7 days with a standard diet. Subsequently, the mice were randomly divided into two groups, the normal control (NC) group (n = 33) and the NAFLD model control (MC) group (n = 150). The MC group mice were intragastrically administered with high-fat emulsion (25% lard oil, 20% sucrose, 10% cholesterol, 10% tween-80, 2% propanediol, 2% sodium deoxycholate, 1% egg yolk power, and 1% propylthiouracil, as a percentage of total weight), and the NC group mice were orally administered with saline every morning for five weeks, and three mice of each group were killed by cervical dislocation per week to confirm NAFLD [27]. In the sixth week, the model group mice were randomly divided into 5 groups: MC as model control group (HFD, 10 mL/kg), PC as positive control group (polyene phosphatidylcholine capsules, PPC, 60 mg/kg), TFC-L as a group treated with low dose of TFC (50 mg/kg), TFC-M as a group treated with intermediate dose of TFC (100 mg/kg), and TFC-H as a group treated with high dose of TFC (200 mg/kg). After regrouping, the experimental process remained unchanged at every morning, with TFC and positive control drugs administered by intragastric administration in every afternoon, from 3:00 p.m. to 5:00 p.m. In parallel, NC was administered in the same amount of normal saline every day. Six mice were killed from each group in the eighth week, the tenth week, and the twelfth week, respectively. Mice were fasted overnight, and body weight was recorded before they were killed. After anesthesia, blood was collected from the orbital plexus, and the mice were killed. The serum was obtained from blood through centrifugation and kept at −80 °C. The liver tissues were stripped, cleaned, and weighed. Then a part of liver and ileum was taken for staining, and the other part was stored at −80 °C for future use. All related procedures and protocols conducted in this experiment were approved by the Animal Care and Welfare Committee of Jiangxi Agricultural University (JXAUA1101). 

### 2.6. Serum and Hepatic Biochemical Analysis

The serum and hepatic levels of TG, TC, ALT, AST, MDA, T-SOD, and GSH-Px were detected by using corresponding commercial kits (Nanjing Jiancheng Bioengineering Institute). The hepatic levels of TNF-α, IL-6, IL-1β, FFA, and ET were determined with ELISA kit (Bost Bioengineering Co., LTD, San Diego, CA, USA). 

### 2.7. Histological Analysis

Liver and ileum tissue samples were immersed into 4% paraformaldehyde, stained with H&E, and then all samples were examined with an optical microscope, according to a reported method with proper modification [27]. 

### 2.8. Western Blotting

The liver samples were homogenized in RIPA lysate and centrifuged at 1200 rpm for 10 min. The BCA assay kit was used to determine the protein content in supernatant. The protein samples with uniform concentration were thoroughly mixed with the loading buffer and denatured in boiling water for 10 min. The proteins in the samples were separated on SDS–PAGE gel and transferred onto a PVDF membrane. The membranes were blocked with 5% non-fat milk and probed with matched primary antibodies overnight and then incubated with secondary antibodies. The protein bands were visualized by using ECL reagent with a Gene Genius Bio-Imaging System. The integral density of protein bands was analyzed by Image J. 

### 2.9. RT-qPCR Analysis

The procedure for detecting the mRNA expression levels of mucin (MUC) 2, MUC4, Zonula occludens-1 (ZO-1), claudin, toll-like receptor 4 (TLR4), and myeloid differentiation factor 88 (MyD88) is based on the reported method, with slight modifications [28]. The total RNAs were firstly extracted from the ileum tissue of mice according to the instructions of the TransZol Up Plus RNA kit. The primers used in this study were listed in Table 1. The expression of target genes was normalized to β-actin gene levels and calculated by using the 2^−ΔΔCt^ method. 

### 2.10. Gut Microbiota Analysis

First, 16S rRNA gene sequencing and bioinformatics analysis were performed as previously described [29]. The specific primers were used to amplify the bacterial 16S rRNA V3/V4 hypervariable region. Samples were sequenced on the Illumina MiSeq PE300 platform (Shanghai Majorbio Bio-Pharm Technology Co., Ltd., Shanghai, China), according to the manufacturer’s agreement. OTU clustering was performed for non-repeating sequences according to 97% similarity. The Chao 1 index and Shannon index were calculated to evaluate the α diversity of samples. Qiime (V1.8.0) was utilized for calculating the beta diversity distance matrix, and the R package was used for the principal co-ordinates’ analysis (PCoA). A correlation analysis was performed for each taxonomic level (phylum and family). 

### 2.11. Statistical Analysis

SPSS Statistics 21 was utilized for statistical analysis. Data were analyzed based on one-way ANOVA and the Duncan test to compare differences between groups. Experimental results were expressed as the means ± standard deviation (M ± SD). Significant differences were set as *p* < 0.05. Origin software (Origin, Farmington, ME, USA) was used for plotting graphs. 

## 3. Results

### 3.1. Identification of Components in TFC

**Figure 1:**
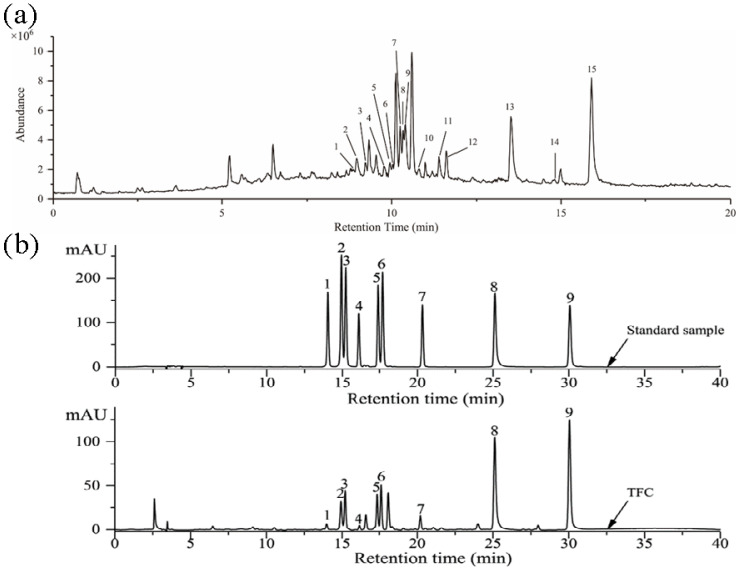
(**a**) The total ions chromatogram of the TFC. Rutin (1); Hyperin (2); Isoquercitrin (3); Kaempferol-3-O-rutinoside (4); Luteolin-5-O-glucoside (5); Quercetin-3-O-xyloside or Quercetin pentoside (6); Kaempferol-3-O-galactoside (7); Astragalin (8); Quercetin-3-O-α-L-rhamnoside (9); Kaempferol-3-O-arabinoside (10); Kaempferol-3-O-acetyl-galactoside (11); Afzelin (12); Quercetin (13); Naringenin (14); Kaempferol (15). (**b**) HPLC chromatographic of standard samples and TFC. Rutin (1); Hyperin (2); Isoquercitrin (3); Kaempferol-3-O-rutinoside (4); Luteolin-5-O-glucoside (5); Astragalin (6); Afzelin (7); Quercetin (8); Kaempferol (9).

Components of TFC were analyzed by LC–MS, and the results were shown in Figure 1a. The identification of individual flavonoid was performed based on comparing and analyzing the retention time, precursor ions, and related fragment ions of each chromatographic peak with the data in the published literature, and fifteen compounds in TFC were preliminarily identified [23,30,31,32,33,34,35,36,37,38]. The results were summarized in Table 2. The HPLC chromatogram of TFC is shown in Figure 1b. By using the spiking test method and comparing with the retention time of the standard, nine flavonoids and their contents in TFC were determined. The quantitative results of TFC flavonoids are shown in Table 3.

### 3.2. Antioxidant Activities of TFC

**Figure 2:**
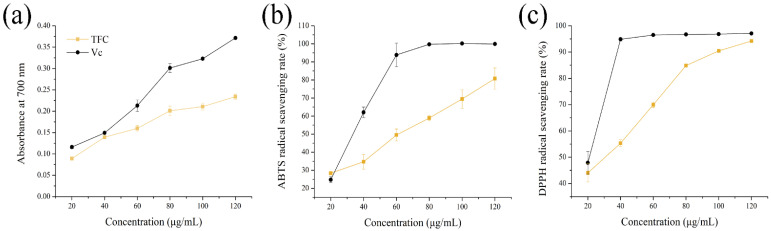
Antioxidant activities of TFC in vitro: (**a**) reducing power, (**b**) ABTS+ scavenging activities (%), and (**c**) DPPH• scavenging activities (%). Vitamin C (Vc) was used as positive control.

Antioxidant activities of TFC were evaluated by ferric reducing power and the scavenging radicals (ABTS+ and DPPH•) in vitro. As shown in Figure 2a, the ferric reducing power of TFC was dose-dependent in the range of 20 to 120 μg/mL. Moreover, TFC showed antioxidant activity in the ABTS+ and DPPH• assays, with great antioxidant capacity, and the IC50 occurred at 54.59 ± 4.12 μg/mL and 28.05 ± 1.78 μg/mL of TFC (Figure 2b,c). This study showed that, although inferior to Vitamin C in an antioxidant capacity, TFC still had commendable antioxidant activity in vitro.

### 3.3. Effects of TFC on Physiological Indices in NAFLD Mice

**Figure 3:**
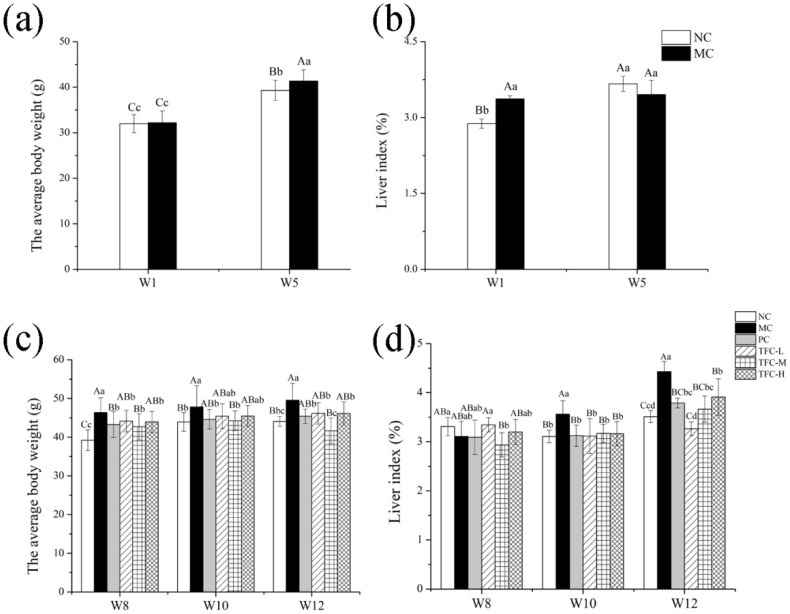
Effects of TFC on weight gain and liver index: (**a**) body weight in the first week and the fifth week; (**b**) liver index in the first week and the fifth week; (**c**) body weight in the eighth week, the tenth week, and the twelfth week; and (**d**) liver index in the eighth week, the tenth week, and the twelfth week. Different letters indicate significant differences: ^abcd^ *p* < 0.05, and ^ABC^ *p* < 0.01.

As shown in Figure 3a, after five weeks of modeling, the weights of mice in the MC group were notably higher than those of the NC group (*p* < 0.01). After TFC intervention, the body weights of mice in the three TFC dose groups were significantly lower than those in the MC group (*p* < 0.05), but there was no dose dependency (Figure 3c). The results showed that TFC could reduce body weight. After ten weeks of modeling, liver indices of the MC group were notably higher than those of the NC group (*p* < 0.01) (Figure 3d). TFC supplementation decreased liver indices. The present results showed that HFD led to body weight gain and liver index augment, which are closely related to the development of NAFLD [39]. Moreover, the results also showed that TFC possesses effective anti-obesity action.

### 3.4. Effects of TFC on Lipid Metabolism and Liver Function in NAFLD Mice

**Figure 4:**
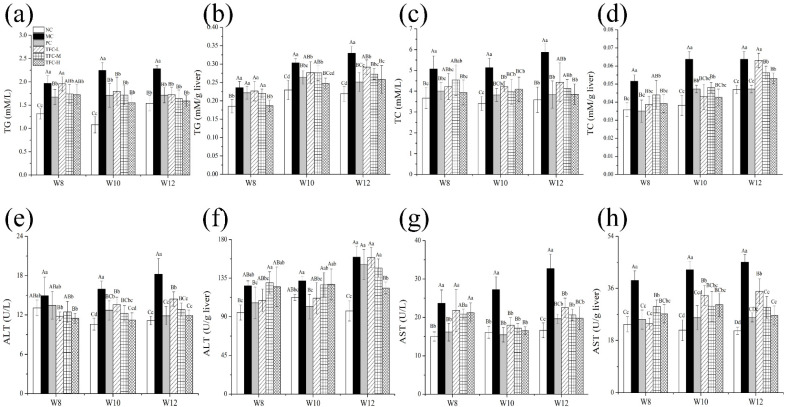
Effects of TFC on lipid metabolism and liver function of NAFLD mice in 8, 10, and 12 weeks: (**a**) serum TG, (**b**) liver TG, (**c**) serum TC, (**d**) liver TC, (**e**) serum ALT, (**f**) liver ALT, (**g**) serum AST, and (**h**) liver AST. Different letters indicate significant differences; ^abcd^ *p* < 0.05, and ^ABCD^ *p* < 0.01.

Compared with the NC group, the levels of TG, TC, AST, and ALT in the serum and liver increased significantly in the MC group (*p* < 0.05) (Figure 4), as was consistent with NAFLD symptoms. Compared with the MC group, the levels of blood lipid and transaminase in the serum and liver showed a significant dose–effect reduction after TFC treatment (*p* < 0.05), and its effect was near to that of positive drugs. The results indicated that TFC regulated lipid metabolism and alleviated abnormal activities of transaminase in NAFLD mice.

### 3.5. TFC Ameliorated Liver Pathological Injuries

**Figure 5:**
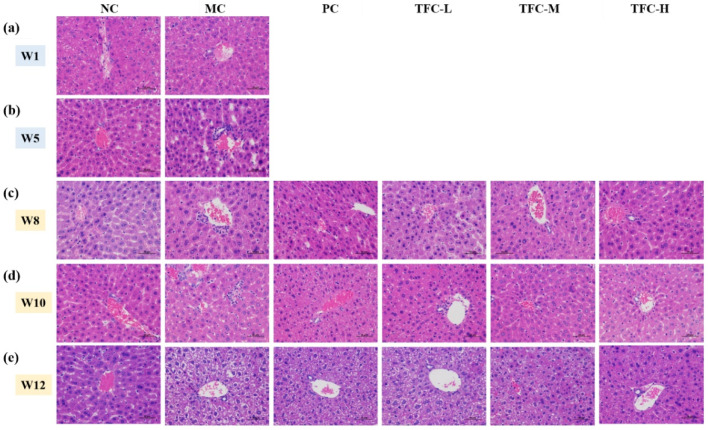
Representative images of liver H&E staining (400×) in different groups of mice. (**a**) Hepatic histomorphology in the first week, (**b**) Hepatic histomorphology in the fifth week, (**c**) Hepatic histomorphology in the eighth week, (**d**) Hepatic histomorphology in the tenth week, (**e**) Hepatic histomorphology in the twelfth week.

As displayed in Figure 5, the NC group showed an intact structure of lobule in the liver. The H&E staining results for model mice in the fifth week showed there were fuzzy cell boundaries and multiple inflammatory foci in the liver (Figure 5b), so that classical cytological features confirmed the successful establishment of the animal model [40,41]. The MC group’s liver pathological damage was more severe compared with that of the NC group as modeling time extended, especially in the twelfth week (Figure 5c–e). In contrast, TFC treatment can effectively relieve the extent of hepatocyte ballooning degeneration and loose cytoplasm. In particular, the liver tissues of the TFC-H group were similar to those of the NC group.

### 3.6. TFC Attenuated Oxidative Stress in NAFLD Mice

**Figure 6:**
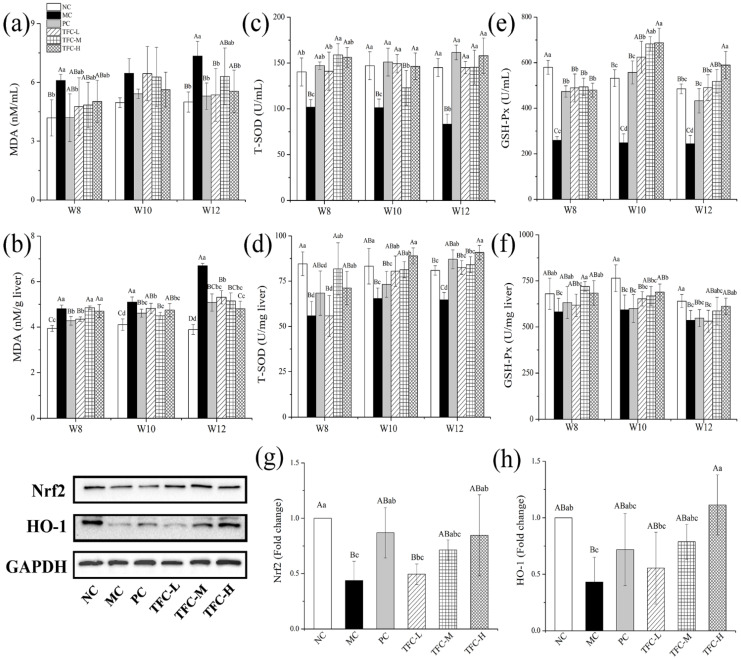
Effects of TFC on oxidative stress in NAFLD mice: (**a**) serum MDA, (**b**) liver MDA, (**c**) serum T-SOD, (**d**) liver T-SOD, (**e**) serum GSH-Px, and (**f**) liver GSH-Px. The protein expression of (**g**) Nrf2 and (**h**) HO-1 in the liver in the twelfth week was detected by Western blotting. Different letters indicate significant differences; ^abcd^ *p* < 0.05, and ^ABCD^ *p* < 0.01.

Compared with the NC group, T-SOD and GSH-Px activities were remarkably decreased, and MDA was overproduced in HFD mice (Figure 6a–f). However, the elevation of the MDA level was greatly declined in mice treated with TFC (Figure 6a,b). Meanwhile, HFD-reduced activities of T-SOD and GSH-Px were significantly elevated in mice treated with TFC (Figure 6c–f). The liver protein expression levels of Nrf2 and HO-1 in the MC group were markedly more downregulated than those of the NC group (Figure 6g–h). This change was significantly reversed by PPC and TFC treatments.

### 3.7. TFC Reduced FFA Level and Alleviated Liver Inflammation in NAFLD Mice

**Figure 7:**
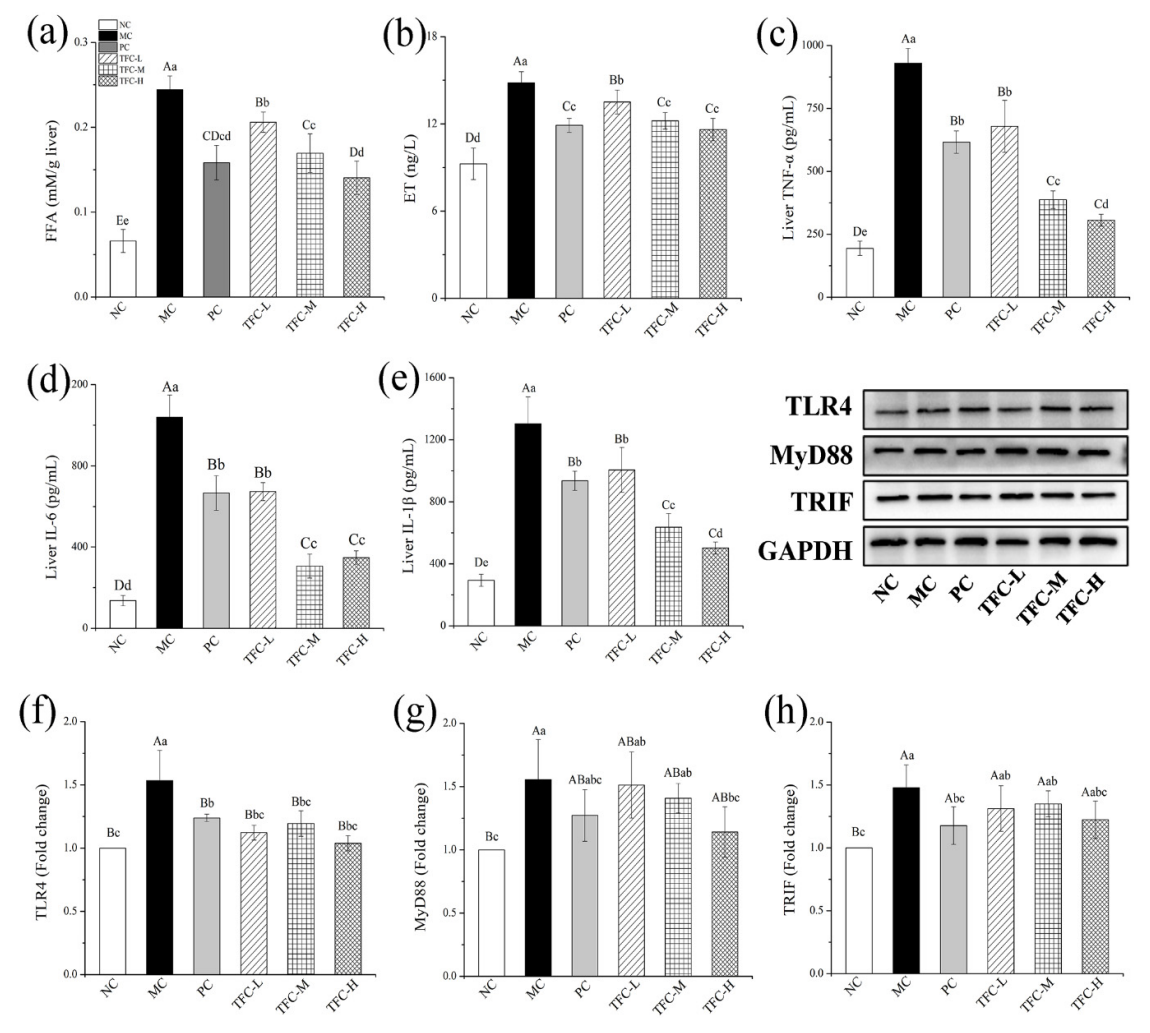
Effects of TFC on FFA level and liver inflammation of NAFLD mice in the twelfth week: (**a**) liver FFA, (**b**) liver ET, (**c**) TNF-α, (**d**) IL-6, and (**e**) IL-1β. The protein expression of TLR4 (**f**), MyD88 (**g**), and TRIF (**h**) in the liver in the twelfth week was detected by Western blotting. Different letters indicate significant differences; ^abcd^ *p* < 0.05, and ^ABCD^ *p* < 0.01.

In the twelfth week, the FFA, ET, TNF-α, IL-6, and IL-1β levels were significantly increased in the MC group as compared with the NC group (*p* < 0.05) (Figure 7a–e). After seven weeks of the TFC intervention, the hepatic levels of FFA, ET, and inflammatory factors of TFC-treated mice were remarkably lower than those of the MC group (*p* < 0.05). The inflammatory factors’ levels in TFC-M and TFC-H groups were lower than those in the PC group, indicating that the anti-inflammatory effect of TFC at medium and high doses was better than PPC.

The effects of TFC on the liver protein expressions of TLR4, MyD88, and TRIF are shown in Figure 7f–h. In the twelfth week, the expression levels of TLR4, MyD88, and TRIF in the MC group were significantly higher than those in the NC group (*p* < 0.01). On the contrary, compared with the MC group, TFC significantly reduced the protein expression levels of TLR4, MyD88, and TRIF (*p* < 0.05), and the improvement effect of the TFC-H group was superior compared to that of the PC group.

### 3.8. TFC Protected Intestinal Barrier Integrity in NAFLD Mice

**Figure 8:**
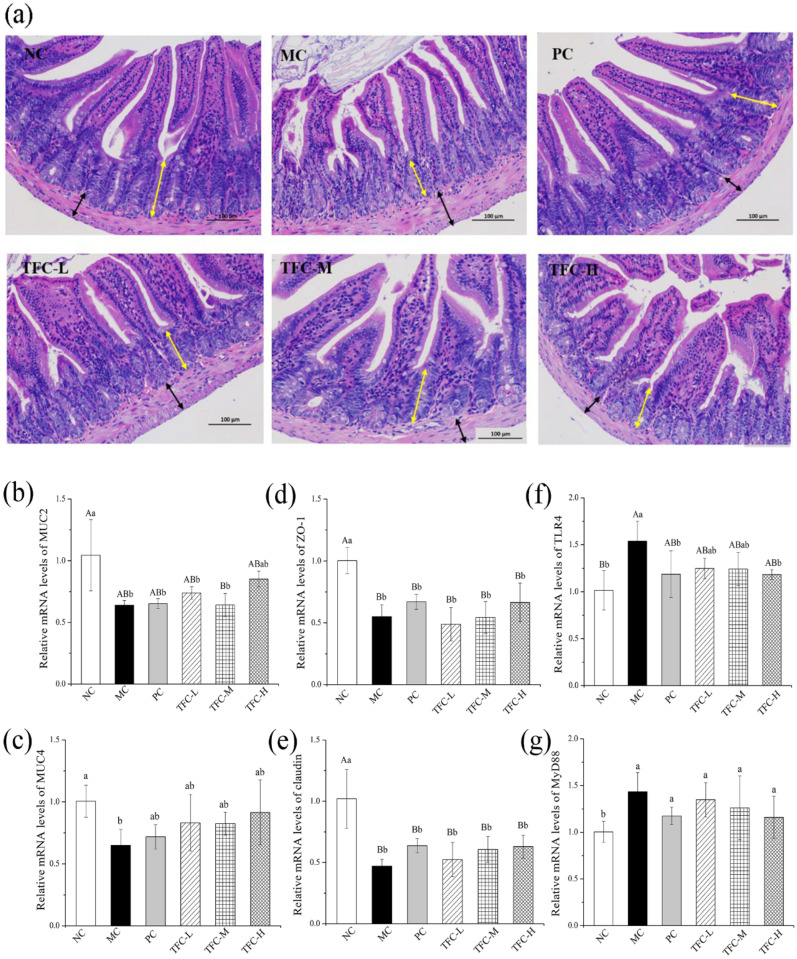
(**a**) Representative images of intestinal tissue H&E staining (200×) in different groups of mice (yellow arrow indicates crypt depth; black arrow indicates muscular thickness) and the mRNA expression of MUC-2 (**b**), MUC-4 (**c**), ZO-1 (**d**), Occludin (**e**), TLR4 (**f**), and MYD88 (**g**) in small intestine. Different letters indicate significant differences; ^ab^ *p* < 0.05, and ^AB^ *p* < 0.01.

As shown in Figure 8a, the intestinal morphology of the NC group was normal. In the MC group, the intestinal tissue showed shortened villi, a shallow crypt depth, and increased musculus thickness. After TFC treatment, the pathological damage of intestinal tissue was significantly relieved. The results showed that TFC can improve intestinal injury in HFD-induced NAFLD mice.

By contrast with the NC, the gene transcription levels of MUC 2, MUC4, ZO-1, and claudin in intestinal tissues were greatly declined in the MC group, while the transcription levels of TLR4 and MyD88 were notably elevated (Figure 8b–g). The TFC treatment upregulated the gene levels of MUC2, MUC4, ZO-1, and claudin and downregulated the gene expressions of TLR4 and MyD88 when compared with the MC group.

### 3.9. TFC Restored Intestinal Microbiome Imbalance in NAFLD Mice

**Figure 9:**
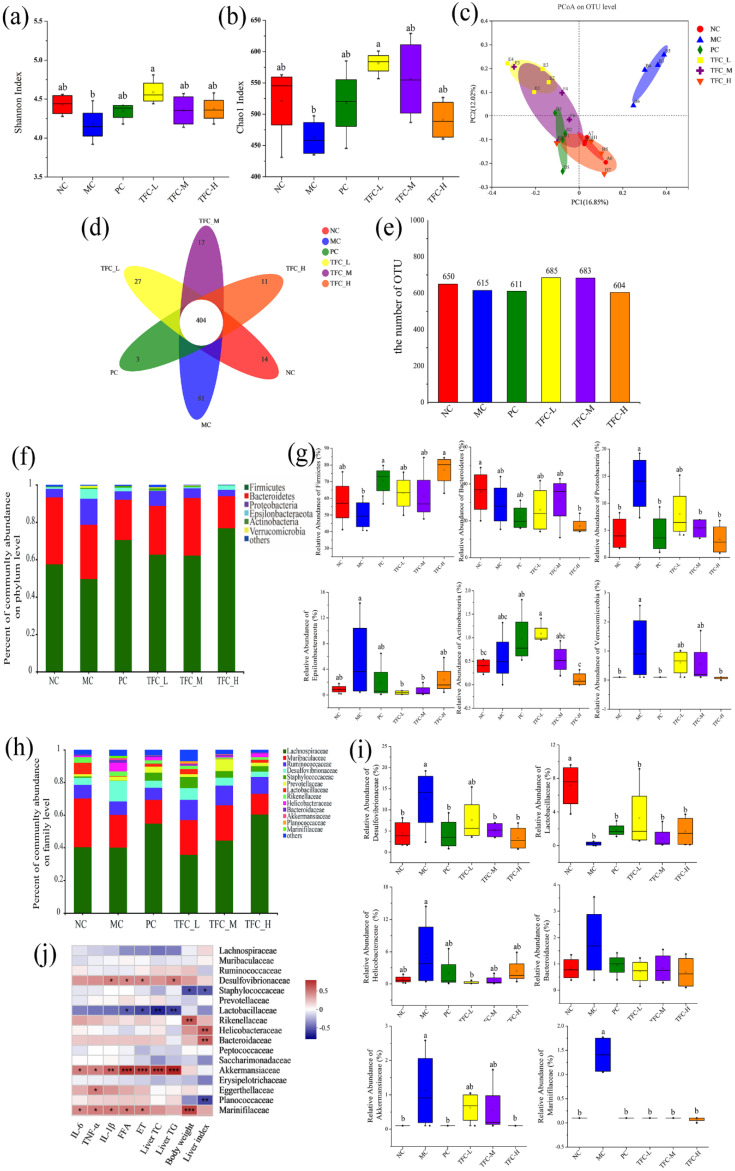
Effects of TFC on intestinal microbial composition and diversity. (**a**) Shannon index, (**b**) Chao1 index, (**c**) PCoA, (**d**) Venn diagram, (**e**) the number of OTU, (**f**) phylum-level abundance, (**g**) relative abundances of the gut microbiota at the phylum levels, (**h**) family-level abundance, and (**i**) relative abundances of the gut microbiota at the family levels. (**j**) Heat map of Spearman’s rank correlation between related indexes of NAFLD and changes of gut microbiota distribution at the family level. Red color indicates positive correlation, and blue color indicates negative correlation; * *p* < 0.05, ** *p* < 0.01, and *** *p* < 0.001. Different letters indicate significant differences; ^abc^ *p* < 0.05.

The results of the Shannon index and Chao 1 index showed that TFC significantly increased the total number and diversity of intestinal microbiota compared with MC group (Figure 9a,b). As shown in Figure 9c, all groups had a distinct clustering of microbiota composition. Compared with the NC group, the HFD significantly changed the composition of gut microbial community in the MC group. The microbial community structure of the TFC-H was more similar to that of the NC group. The number of OTUs in the TFC-L and TFC-M groups was significantly increased compared with the NC group (Figure 9d,e). At the phylum level, the relative abundance of *Firmicutes* and *Bacteroidetes* in the MC group decreased, while the relative abundance of *Proteobacteria* and *Verrucomicrobia* significantly increased. This change was significantly reversed in the TFC treatment group (Figure 9f,g). At the level of the family, 12 families were identified (Figure 9h,i). The relative abundance of *Desulfovibrionaceae*, *Helicobacteraceae*, *Bacteroidaceae*, *Akkermansiaceae*, and *Marinifilaceae* in the MC group was increased, while that of *Lactobacillaceae* was decreased. In addition, TFC treatment changed the relative abundance of *Desulfovibrionaceae*, *Helicobacteraceae*, *Bacteroidaceae*, *Akkermansiaceae*, *Marinifilaceae*, and *Lactobacillaceae* toward the NC group.

The Spearman rank correlation coefficient was used to analyze the association between the gut microbiota at the family level and related indicators of inflammatory and lipid metabolism in the liver (Figure 9j). The levels of TG and TC in the liver were negatively correlated with *Lactobacillaceae*, but positively correlated with *Akkermansiaceae*. There was a positive correlation between *Helicobacteraceae* and *Bacteroidaceae* with the liver index. The levels of FFA, ET, and related inflammatory factors such as IL-6, IL-1β, and TNF-α in the liver are positively correlated with *Akkermansiaceae*, *Desulfovibrionaceae*, and *Marinifilaceae*, but negatively correlated with *Lactobacillaceae*.

## 4. Discussion

With the change of people’s lifestyle, health problems such as obesity, hyperlipidemia, and HFD-induced NAFLD are becoming more and more serious. As the pathogenesis of NAFLD is not fully understood, there are currently no drugs approved for the treatment of NAFLD. According to previous studies, the treatment of NAFLD with natural products in regard to lipid metabolism, oxidative stress, and inflammation is considered to be a promising treatment prospect [42,43].

Lipid metabolism disorder is one of the characteristics of metabolic syndrome and has been proved to be closely related to NAFLD. Previous studies have shown that the pathogenesis of NAFLD relates to a manifold process, and adiposity leads to excessive FFAs, which are taken up by various transporters to be delivered into the liver through the blood circulation [34]. Firstly, the increased TG accumulation caused by the esterification of FFA eventually leads to the formation of lipid droplets in hepatocyte, and this is the early pathological feature of NAFLD [44,45]. Meanwhile, hepatobiliary steroid homeostasis disorder and hepatic free cholesterol accumulation affect the pathogenesis of NAFLD [46]. The increased lipid accumulation is a major factor resulting in hepatocyte destruction, leading to the elevation of AST and ALT levels, which are markers of liver damage [47]. Quercetin, as one of the main components of TFC, has a strong antioxidant stress effect and can inhibit hepatocyte apoptosis and inflammation [48]. Studies have confirmed that quercetin can reduce TG levels in circulating supply and endogenous synthesis; prevent excessive TG and its components from deposition in non-adipose tissues, such as the liver; and reduce TC, ALT, and AST levels, thereby improving NAFLD [49]. In this study, the HFD led to the elevation of AST and ALT levels in the serum and liver of mice, and the pathological changes, such as edema and balloon-like degeneration, were found in the liver. The larger liver weight may be due to more fat accumulation and liver edema in MC group. TFC treatment improved lipid metabolism, reduced abnormal activity of liver and serum transaminase in NAFLD mice, reversed liver cell damage caused by HFD, and reduced liver index. The beneficial effects of TFC may be closely related to its high levels of quercetin.

Oxidative stress is a crucial step in the course of NAFLD. FFA in the liver can be utilized by β oxidation, but excessive FFA can lead to an increase of free oxygen in the body that induces mitochondrial dysfunction and causes oxidative stress [50]. As the antioxidant system is damaged, excessive lipid peroxides MDA is produced and leads to cell damage [51]. SOD and GSH-Px are the main enzymes of scavenging free radicals, and they are involved in scavenging superoxide and intermediate products produced in the oxidation process [24]. In our study, we found that TFC can regulate the balance of free radicals by increasing the activity of antioxidant enzymes and inhibiting lipid peroxidation and improved HFD-induced oxidative stress. The Nrf2/HO-1 pathway is closely related to oxidative stress, and the expression levels of the Nrf2/HO-1 pathway reflect the degree of oxidative stress [52]. Previous studies have shown that corn peptides can provide potential prevention and adjuvant treatment for NAFLD by regulating Nrf2/HO-1 pathway expression and inhibiting oxidative stress [53]. Our results are consistent with previous studies that have shown that TFC can regulate the expression of Nrf2 and HO-1 proteins and ultimately achieve the effect of alleviating NAFLD.

Increasing evidence has pointed out that the gut–liver axis closely relates to the occurrence of NAFLD. Studies have shown that HFD can disrupt the intestinal barrier and increase intestinal permeability, allowing ET to enter the liver through the circulation [54]. ET is an inflammatory inducer that induces liver inflammation by activating the TLR4/MyD88 pathway and promotes the release of inflammatory factors in liver tissues, and this, in turn, could cause further intestinal barrier dysfunction [55]. Kaempferol, another active compound existing in TFC, has been proved to inhibit the occurrence and development of NAFLD through multi-target action; for example, it suppresses inflammation, improves insulin resistance, and reduces oxidative stress [56]. The results of this study showed that TFC can reduce the level of inflammatory factors and ET in the liver and upregulate the expression of inflammatory-pathway-related proteins, thus improving the inflammatory response of the liver. The anti-inflammatory effect of TFC may be closely related to its rich content of kaempferol. Meanwhile, TFC regulates the expression levels of genes associated with the intestinal barrier, thereby preventing bacteria and ET from entering the circulation and liver. The changes of intestinal-barrier-related gene expression levels were consistent with the results of small intestine section, suggesting that TFC has the effect of improving intestinal barrier. Through the above effects, TFC regulated the gut–liver axis to achieve the effect of alleviating NAFLD.

The microbial barrier plays an important role in intestinal health, and gut microbiota is the main influencing factor of microbial barrier. Mounting evidence has shown that the severity of NAFLD is related to intestinal dysbiosis, and there is a bidirectional relationship between them [8]. Studies have shown that an HFD has induced intestinal microbiota imbalance in NAFLD mice, resulting in intestinal dysfunction [57]. In contrast, transplanting gut microbiota from NAFLD patients into HFD-fed mice increased liver fat deposition and inflammation in the mice, indicating that intestinal microbiota could also affect the development of NAFLD [11]. Our data revealed that TFC changed the composition of gut microbiota in NAFLD mice. TFC increased the relative abundance of *Firmicutes* and *Bacteroidetes* and decreased the relative abundance of *Proteobacteria*. On the one hand, TFC reduces the relative abundance of pathogenic bacteria. The family *Desulfovibrionaceae* within the phylum *Proteobacteria* is an essential group producing ET, which is vital to regulate liver and intestinal inflammatory response [58,59]. *Helicobacteraceae* is one of the most common bacterial pathogens in human body, mainly causing gastritis and gastrointestinal ulcer [60]. Some studies have suggested that *Helicobacteraceae* infection may have a potential function in hepatocyte degeneration in NAFLD [61]. In this study, TFC reduced the relative abundance of *Desulfovibrionaceae* and *Helicobacteraceae*, which may be beneficial for improving the intestinal barrier, relieving inflammation and fat deposition in liver, and ultimately alleviating NAFLD. The correlation analysis between the gut microbiota and NAFLD phenotype also confirmed that *Desulfovibrionaceae* and *Helicobacteraceae* were positively correlated with inflammation and lipid-metabolism-related indicators in liver. On the other hand, TFC increases the relative abundance of probiotics. *Lactobacillaceae* can attenuate the progression of NAFLD by reducing TC and steatosis [62]. An HFD reduced the relative abundance of *Lactobacillaceae* in mice, while TFC reversed this change, and the correlation analysis showed that *Lactobacillaceae* was negatively correlated with TC, TG, FFA, and ET levels in the liver. The results indicated that TFC reduces TC and TG levels and liver steatosis in mice, which may be through regulating the proportion of probiotics. In our experiment, TFC protected the integrity of the intestinal barrier, restored the gut microbiota, and alleviated NAFLD in mice. Compared with the three doses, high-dose TFC showed a better effect in alleviating NAFLD. However, which substances in TFC play a major role in alleviating NAFLD and its mechanism need to be further studied.

## 5. Conclusions

In summary, TFC can improve HFD-induced NAFLD through the gut–liver axis. TFC reduces the levels of TG, TF, and FFA by reducing the accumulation of fat around the body, so as to relieve the body’s lipid metabolism disorder and oxidative stress. Moreover, TFC restores the integrity of the intestinal barrier, improves the imbalance of intestinal flora, reduces intestinal permeability, and prevents the migration of intestinal flora and ET, thus reducing inflammatory response and improving the pathological conditions of liver and intestinal tissues. Summarily, our data suggest that TFC has the potential to prevent or treat NAFLD.

## Figures and Tables

**Table 1:** Primer sequences of RT-qPCR.

Primer Name	Forward	Reverse
ZO-1	5′-CCAGAGCCTCAGAAACCTCA-3′	5′-GCAGGAAGATGTGCAGAAGG-3′
Claudin	5′-ACGGTCTTTGCACTTTGGTC-3′	5′-GGGAGAGGAGAAGCACAGTT-3′
MUC-2	5′-TGTGGTCTGTGTGGGAACTT-3′	5′-GCTTACATCTGGGCAAGTGG-3′
MUC-4	5′-AACTCCTTCAGCCTCCTCAC-3′	5′-GCTGTTGTGTGTCCTGAGTC-3′
TLR4	5′-TAGCCATTGCTGCCAACATC-3′	5′-CCTCAGCAGGGACTTCTCAA-3′
MyD88	5′-GCATGGTGGTGGTTGTTTCT-3′	5′-AACCGCAGGATACTGGGAAA-3′
β-actin	5′-CCAGCCTTCCTTCTTGGGTA-3′	5′-CAATGCCTGGGTACATGGTG-3′

**Table 2:** Negative-ion ESI mass spectra of the main constituents of the TFC.

No.	RT (min)	Molecular ion (m/z)	Fragment ions (m/z)	Formula	Proposed Compounds
1	8.888	609	300	C_27_H_30_O_16_	Rutin
2	8.986	463	301; 151	C_21_H_20_O_12_	Hyperin
3	9.218	463	301/300; 271	C_21_H_20_O_12_	Isoquercitrin
4	9.788	593	285	C_27_H_30_O_15_	Kaempferol-3-O-rutinoside
5	9.935	447	285/284; 151	C_21_H_20_O_11_	Luteolin-5-O-glucoside
6	10.049	433	301/300	C_20_H_18_O_11_	Quercetin-3-O-xyloside or Quercetin pentoside
7	10.239	447	285/284	C_21_H_20_O_11_	Kaempferol-3-O-galactoside
8	10.350	447	285/284	C_21_H_20_O_11_	Astragalin
9	10.414	447	301/300; 151	C_21_H_20_O_11_	Quercetin-3-O-α-L-rhamnoside
10	10.812	417	285	C_20_H_18_O_10_	Kaempferol-3-O-arabinoside
11	11.407	489	285/284	C_23_H_22_O_12_	Kaempferol-3-O-acetyl-galactoside
12	11.618	431	285	C_21_H_20_O_10_	Afzelin
13	13.499	301	151	C_15_H_10_O_7_	Quercetin
14	14.801	271	151	C_15_H_12_O_5_	Naringenin
15	15.917	285	227	C_15_H_10_O_6_	Kaempferol

**Table 3:** Components and contents of flavonoids from TFC.

Peak	Compounds	RT (min)	Calibration Curve	R^2^	TFC (μg/mg)	LOD	LOQ
1	Rutin	13.970	Y = 10.714x − 2.5708	0.9986	2.612 ± 0.275	0.070	0.235
2	Hyperin	14.919	Y = 26.761x − 1.4147	0.9986	5.308 ± 0.093	0.032	0.105
3	Isoquercitrin	15.194	Y = 13.457x − 9.2557	0.9985	13.301 ± 0.072	0.063	0.209
4	Kaempferol-3-O-rutinoside	16.192	Y = 9.640x + 7.5889	0.9999	1.321 ± 0.097	0.094	0.314
5	Luteolin-5-O-glucoside	17.360	Y = 11.221x + 2.8667	0.9982	13.479 ± 0.041	0.080	0.265
6	Astragalin	17.621	Y = 11.88x + 55.128	0.9936	13.959 ± 0.025	0.084	0.279
7	Afzelin	20.211	Y = 24.013x − 352.22	0.9909	9.781 ± 0.033	0.044	0.145
8	Quercetin	25.117	Y = 13.745x − 21.717	0.9999	45.489 ± 1.206	0.080	0.267
9	Kaempferol	30.047	Y = 13.554x – 3.8523	0.9993	51.293 ± 0.715	0.088	0.294

## Data Availability

The data presented in this study are available upon request from the corresponding author. The data are not publicly available due to privacy.
